# Differential Responses of Pattern Recognition Receptors to Outer Membrane Vesicles of Three Periodontal Pathogens

**DOI:** 10.1371/journal.pone.0151967

**Published:** 2016-04-01

**Authors:** Jessica D. Cecil, Neil M. O’Brien-Simpson, Jason C. Lenzo, James A. Holden, Yu-Yen Chen, William Singleton, Katelyn T. Gause, Yan Yan, Frank Caruso, Eric C. Reynolds

**Affiliations:** 1 Oral Health Cooperative Research Centre, Melbourne Dental School, Bio21 Institute, The University of Melbourne, Melbourne, Victoria, Australia; 2 Department of Chemical and Biomolecular Engineering, The University of Melbourne, Parkville, Victoria, Australia; Oregon Health & Science University, UNITED STATES

## Abstract

Highly purified outer membrane vesicles (OMVs) of the periodontal pathogens, *Porphyromonas gingivalis*, *Treponema denticola* and *Tannerella forsythia* were produced using tangential flow ultrafiltration, ultracentrifugation and Optiprep density gradient separation. Cryo-TEM and light scattering showed OMVs to be single lipid-bilayers with modal diameters of 75 to 158 nm. Enumeration of OMVs by nanoparticle flow-cytometry at the same stage of late exponential culture indicated that *P*. *gingivalis* was the most prolific OMV producer. *P*. *gingivalis* OMVs induced strong TLR2 and TLR4-specific responses and moderate responses in TLR7, TLR8, TLR9, NOD1 and NOD2 expressing-HEK-Blue cells. Responses to *T*. *forsythia* OMVs were less than those of *P*. *gingivalis* and *T*. *denticola* OMVs induced only weak responses. Compositional analyses of OMVs from the three pathogens demonstrated differences in protein, fatty acids, lipopolysaccharide, peptidoglycan fragments and nucleic acids. Periodontal pathogen OMVs induced differential pattern recognition receptor responses that have implications for their role in chronic periodontitis.

## Introduction

A common feature of Gram-negative bacteria is the biogenesis of outer membrane vesicles (OMVs). Once regarded as cell debris or microscopy artefacts, these spherical membrane structures (20–300 nm in diameter) are now thought to play a significant role in microbial virulence [1]. OMVs are closed proteoliposomes composed of outer membrane lipopolysaccharide, lipids, lipoproteins/peptides, porins and receptors, adhesins and enzymes as well as periplasmic components including peptidoglycan fragments [1]. A number of functions have been ascribed to OMVs, including regulation of stress responses, quorum sensing, horizontal gene transfer, co-aggregation of bacteria and biofilm formation as well as reducing toxic components in the environment [2, 3]. In addition, OMVs are highly immunogenic and are considered to enhance pathogenicity by triggering the release of proinflammatory and immunoregulatory cytokines, inducing neutrophil migration and recruitment and disrupting tight junctions in epithelial cell mono-layers [2, 4, 5]. Due to their size, adhesive and proteolytic properties, a prescribed role of OMVs in the virulence of bacteria is that of a novel secretion system that delivers bacterial virulence factors deep into host tissues, stimulating and dysregulating the host immune response and competing with whole cells for contact with host immune molecules and cells, thereby increasing bacterial survival [5].

Chronic periodontitis is an inflammatory disease of the supporting tissues of the teeth, leading to eventual tooth loss, and has been associated with an increased risk of cardiovascular diseases, adverse pregnancy outcomes, respiratory infections, rheumatoid arthritis and diabetes mellitus [6, 7]. A pathogenic hallmark of the disease is an increase in the Gram-negative population of the subgingival plaque biofilm; in particular, proportional increases in the proteolytic species *Porphyromonas gingivalis*, *Treponema denticola* and *Tannerella forsythia* [8, 9]. *P*. *gingivalis* has been shown to shed large numbers of OMVs of approximately 50–70 nm in diameter that are enriched in virulence factors like lipopolysaccharide (LPS), muramic acid, fimbriae and gingipains (Arg- and Lys-specific proteolytic enzymes) to the exclusion of other outer membrane proteins [10–13].

*P*. *gingivalis* OMVs are reported to be highly inflammatory and induce infiltration of neutrophils in connective tissue [4], nitric oxide production [14], foam cell formation in macrophages [15], and have the ability to invade oral epithelial cells [16]. *T*. *denticola*, a spirochete closely associated with *P*. *gingivalis* in periodontitis, also releases extracellular vesicles approximately 50–100 nm in diameter from its outer sheath [17]. *T*. *denticola* OMVs possess protein and proteolytic activity patterns very similar to that of the isolated outer sheath [17]. Several important virulence factors have been identified in *T*. *denticola* OMVs including lipooligosaccharide (LOS) and Major Sheath Protein (Msp) [18]. *T*. *denticola* OMVs have been shown to disrupt tight junctions in Hep-2 epithelial cell monolayers, which is proposed to facilitate the penetration of underlying tissues [5]. Recently, a *T*. *forsythia* OMV preparation was shown to induce pro-inflammatory cytokine release from macrophage and periodontal fibroblast cell lines [19].

Tissue destruction in chronic periodontitis has been attributed to various virulence factors released from these periodontal pathogens that diffuse from the subgingival plaque biofilm accreted on the tooth root into the subjacent connective tissue which dysregulate the immune response and induce a localised chronic inflammation [20]. Hence OMVs containing these virulence factors are likely to have a major role in disease. Pattern Recognition Receptors (PRRs) expressed by host immune cells, including epithelial cells, are key molecules in the induction of a local immune response. While *P*. *gingivalis* OMVs have been linked to inflammation, the PRRs activated by OMVs, including Toll-like receptors (TLRs) and NOD-like receptors (NLRs) have yet to be identified. Furthermore, the involvement of *T*. *denticola* and *T*. *forsythia* OMVs in inflammation and disease progression is yet to be elucidated. Hence, determining the specific PRRs activated by OMVs released by the periodontal pathogens may provide mechanistic insight into the important role of OMVs in chronic periodontitis.

Previous studies have utilised several methods for the isolation of OMVs, the most common being the removal of whole cells from culture media by low-speed centrifugation and filtration followed by ultracentrifugation [17, 21]. Although effective at recovering OMVs, the ultracentrifugation step also sediments a variety of secreted and medium-derived proteins, cellular fragments and other contaminants with the OMVs [22]. The contaminants may affect the host immune response and thus should be removed from the preparations in order to determine the true response to OMVs. [23, 24]

A further issue in comparing the biological responses induced by OMVs isolated from different bacterial species or under different growth conditions is accurately standardising their concentration. Current methods to estimate the yield of OMVs use either the weight of the OMV pellet or protein content [25, 26]. However, OMV composition can be affected by growth conditions and contamination. Furthermore the size and consequent surface area/volume of the OMVs may be important in terms of interaction of OMVs with host cell receptors. Hence to attribute and compare the immune responses induced by OMVs, removal of non-OMV associated material and standardizing OMV preparations on the number of vesicles would improve our understanding of the role of OMVs in disease. Recently, high sensitivity flow cytometry was used to enumerate eukaryotic cell-derived vesicles/exosomes [27, 28] and this technique can also be applied to bacterial OMVs [29].

In the present study procedures were developed for the isolation and enumeration of highly purified OMVs from *P*. *gingivalis*, *T*. *denticola* and *T*. *forsythia*. Using purified OMVs we determined their composition and their ability to stimulate a variety of PRRs and demonstrated differential responses to OMVs from the three periodontal pathogens that have implications for their role in chronic periodontitis.

## Materials and Methods

### Bacterial Cultures and Growth Conditions

All bacterial strains were obtained from the Melbourne Dental School culture collection and maintained as described previously [13, 30, 31] and as described in the S1 Experimental Procedures.

### Isolation of Outer Membrane Vesicles

For OMV purification, bacteria were grown to late exponential phase, determined by optical density and defined as the last period of exponential cell growth before the inhibitory effects of the stationary phase as observed on a growth curve. Whole bacterial cells were removed by centrifugation at 8,000 x g for 30 min at 4°C (using a F10BCI-6x500g rotor installed in an Avanti J-25I Centrifuge, Beckman Coulter, NSW, Australia). The collected supernatant was filtered through a 0.22 μm filter (Merck-Millipore, Victoria, Australia) and then concentrated through a 100-kDa filter using a tangential flow filtration Minimate TFF System (PALL Life Sciences, Victoria, Australia) according to the manufacturer’s instructions. The collected concentrate was centrifuged at 100,000 x g for 2 hours at 4°C (using a JA-30.50 Ti fixed angle rotor installed in an Avanti J-30I Centrifuge, Beckman Coulter, NSW, Australia) to yield a crude OMV preparation. The crude OMVs were then resuspended in 800 μL HEPES buffer (50 mM HEPES, 150mM NaCl, pH 6.8).

Highly purified OMVs were prepared using OptiPrep^™^ (60% w/v iodixanol in water, Sigma-Aldrich NSW, Australia) density gradient centrifugation; whereby crude OMV preparation was resuspended in 3 mL HEPES buffer containing 45% w/v iodixanol and placed in an Ultra-Clear^™^, 14 mL, 14 x 95 mm tube (Beckman Coulter, NSW, Australia). A discontinuous iodixanol gradient was achieved by layering successive 1.5 mL of HEPES buffer containing 40%, then 35%, 30%, 25%, 20% and 15% w/v iodixanol. After centrifugation at 150,000 x g for 16 hours (48 hours for *T*. *forsythia* and *T*. *denticola*) at 4°C (using a SW40 Ti rotor installed in an Optima L-80XP Ultracentrifuge, Beckman-Coulter, NSW, Australia) eight 1.5 mL gradient fractions were collected by pipette from top to bottom of the density gradient solution. Gradient fractions containing purified OMVs were identified by protein analysis using a Qubit 2.0 Fluorometer according to the manufacturer’s instructions (Life Technologies, NSW, Australia). OMV protein profiles were determined by SDS-PAGE analysis using conditions previously described [32] using NuPAGE^®^ Novex 4–12% Bis-Tris gels with MOPS running buffer (Life Technologies, NSW Australia) and SimplyBlue or SyproRuby (Life Technologies, NSW Australia) protein staining.

Fractions containing the purified OMVs were pooled and washed with 0.01 M phosphate buffered saline (PBS, Sigma-Aldrich, NSW, Australia), pH 7.4 at 150,000 x g for 2 hours at 4°C (using a SW40 Ti rotor installed in an Optima L-80XP Ultracentrifuge, Beckman-Coulter, NSW, Australia) and resuspended in 0.22 μm filtered 0.01 M PBS and stored at 4°C for short term storage (<14 days) and -80°C for long term storage (>14 days).

### Transmission Electron Microscopy (TEM)

Purified OMVs were washed twice in 0.22 μm filtered 0.01 M PBS at 150,000 x g for 2 hours at 4°C. Cryo-transmission electron microscopy was performed using an FEI Tecnai G2 F30 instrument (FEI company) as previously described [33].

### Dynamic Light Scattering (DLS)

Dynamic light scattering (DLS) measurements were performed on a Malvern high performance (HPPS) with a He−Ne laser (633 nm) at an angle of 173°. Data was collected from 100 μL of PBS or HEPES suspended OMVs by twenty acquisitions at 25°C.

### Outer Membrane Vesicle Enumeration

Purified OMVs (100 μL) were washed in 0.01 M PBS and recovered by centrifugation at 150,000 x g for 2 hours at 4°C. Washed OMVs were stained with either; FITC (Life Technologies, USA), Alexa Fluor 488 (Life Technologies, USA), Syto 9 Green Fluorescent Nucleic Acid Stain (Life Technologies, USA), FM 4 64 (Life Technologies, USA), PKH-26 Red Fluorescent Cell Linker (Sigma-Aldrich NSW, Australia) or PKH-67 Green Fluorescent Cell Linker (Sigma-Aldrich NSW, Australia) as per the manufacturer’s instructions. Samples were washed in 0.01 M PBS and recovered by centrifugation at 150,000 x g for 2 hours at 4°C. Directly following staining, samples were re-suspended in 0.1% v/v Tween 20 buffer to reduce OMV aggregation and counted using an Apogee A50-Micro Flow Cytometer calibrated for flow and event rates with Apogee Flow Systems Calibration Beads (Calibration Bead Mix, 110 and 500 YG fluorescent and 180, 240, 300, 590, 880, 1300nm). The OMV sample (150 μL) was analysed at a flow rate of 30 μL/min. Critical steps in OMV enumeration were; 1, the use of a 110 nm fluorescent bead within the size range of the OMV samples to set the flow and event rates of the cytometer; 2, labelling the OMVs with a dye that fluoresced in the same channel as the flow beads and 3, re-suspending OMVs, post labelling, in 0.1% v/v Tween 20 buffer to reduce OMV aggregation. Using these criteria we were able to attribute event rate to OMV counts without adjusting for size and volume discrepancies between flow bead and OMV. Enumeration of whole bacterial cells was determined by flow cytometry using LIVE/DEAD^®^ *Bac*Light^™^ Bacterial Viability and Counting Kit (Life Technologies Pty Ltd, NSW, Australia) and a Cell Lab Quanta SC Flow Cytometer (Beckman Coulter).

### Haemagglutination and Arg-x-specific and Lys-x-specific proteinase Assays

Haemagglutination and proteinase activity assays were performed as we have as described previously [20] and as described in S1 Experimental Procedures.

### Lipopolysaccharide, Fatty Acid, Nucleic Acid and Peptidoglycan Assays

The analyses of OMVs for lipopolysaccharide, fatty acids, nucleic acids and peptidoglycan were performed as described in S1 Experimental Procedures.

### Toll-like and NOD-like Receptor Activation Assays

The activation of the Toll-like receptors (TLR2, TLR4, TLR7, TLR8, TLR9) and NOD-like receptors (NOD1 and NOD2) were assayed using HEK-Blue cell lines as described in S1 Experimental Procedures.

## Results

### Isolation of Highly Purified OMVs

All bacteria were grown in batch culture and OMVs harvested at the same stage of late exponential growth. After an initial low-speed centrifugation step to remove whole cells from the culture media, the pellet was examined by Transmission Electron Microscopy (TEM) and found to contain very few OMVs which were either attached to bacteria or very large, > 0.3 μm in diameter (S1 Fig). A tangential flow filtration step was used to reduce the volume of culture medium supernatant prior to ultracentrifugation and resulted in a slight decrease (8.2 ± 5.2%) in total protein concentration. Centrifugation for two hours (100,000 x g) was found to be optimal in isolating a crude OMV preparation.

**Fig 1:**
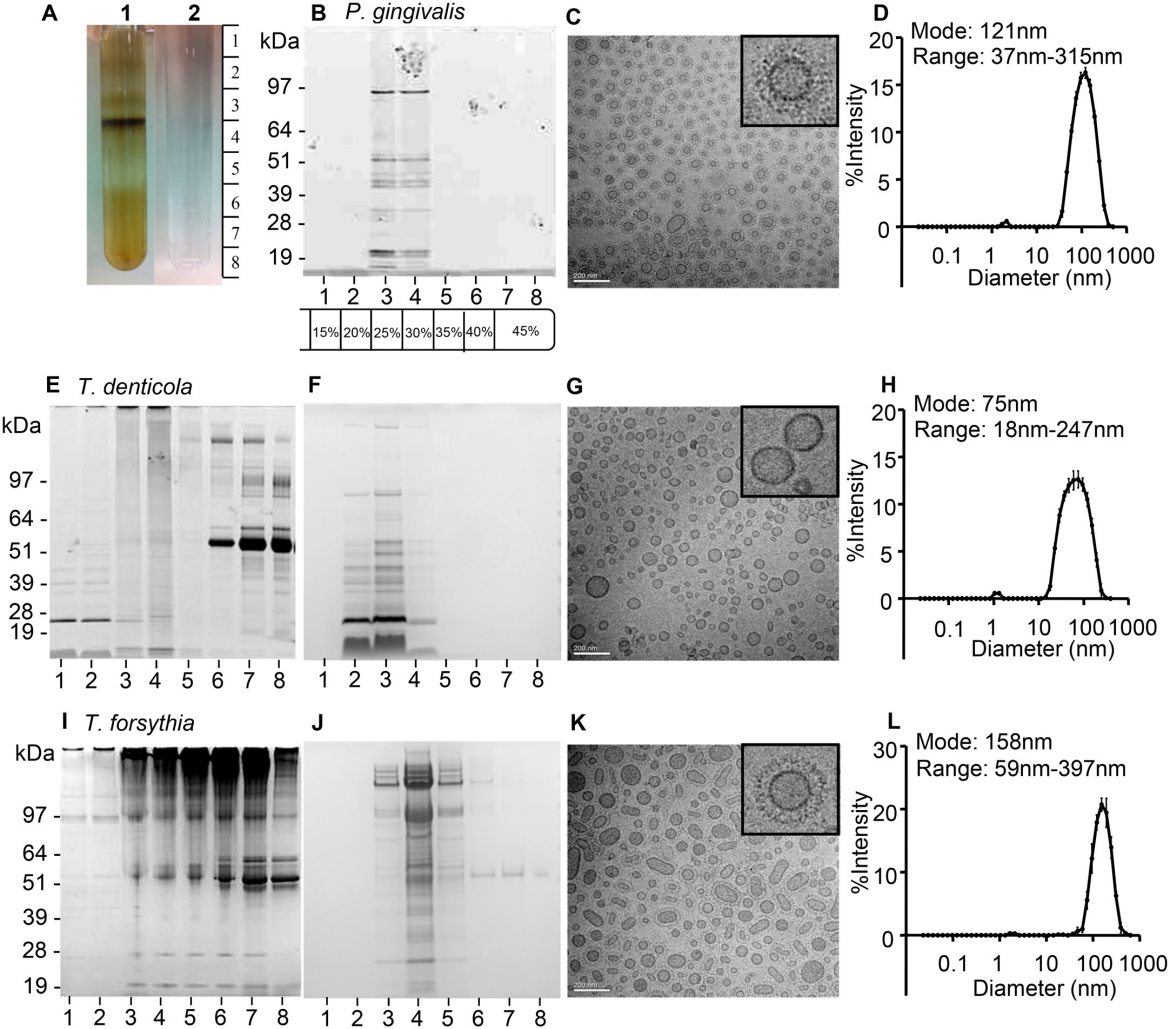
Isolation of highly purified *P*. *gingivalis*, *T*. *denticola* and *T*. *forsythia* OMVs. OptiPrep density gradient centrifugation of a crude *T*. *forsythia* OMV preparation (A1) separated non-OMV associated material (bottom of density gradient) from highly purified OMVs isolated at a higher density. A blank OptiPrep density gradient is shown in A2. SDS-PAGE of *P*. *gingivalis* (B), *T*. *denticola* (E F) and *T*. *forsythia* (I J) Optiprep density gradient fractions taken in eight 1.5 mL gradient fractions from top to bottom following centrifugation. Gradient fractions were subjected to SDS-PAGE and protein bands visualized by SimplyBlue or SyproRuby staining. Molecular mass markers (Novex SeeBlue Plus2 Prestained Standard) are indicated in kDa. OMVs were observed using transmission electron microscopy (TEM) (C G K). Vesicle size and range was further confirmed using dynamic light scattering (DLS) on pooled and washed OMV containing gradient fractions (D H L).

To remove cell debris, protein and lipid aggregates and other non-OMV associated material, crude OMVs were subjected to OptiPrep density gradient separation. A 45% to 15% w/v iodoxanol/HEPES gradient resulted in optimal separation of OMVs from non-OMV associated material (Fig 1A). For *P*. *gingivalis* OMVs, centrifugation at 150,000 x g for 16 hours resulted in OMVs reaching a density equilibrium point at gradient fractions 3 and 4, as determined by SDS-PAGE gel analysis (Fig 1B).

To determine OMV size, purity and structure, purified OMVs were examined by Cryo TEM and dynamic light scattering (DLS). *P*. *gingivalis* OMVs were found to be spherical in shape and consisted of a single lipid-bilayer with an electron dense surface layer (EDSL) of approximately 20 nm. No inner membrane or cellular fragments were observed by TEM in the purified OMV preparation. *P*. *gingivalis* OMVs were found to have a size distribution of 50-150nm, as determined by Cryo-TEM (Fig 1C) and a fully hydrated size range of 37–315 nm with a modal vesicle diameter of 121 nm as determined by DLS (Fig 1D).

On application of the above method to the isolation of purified *T*. *denticola* and *T*. *forsythia* OMVs, major protein contaminants of 65 kDa and 66 kDa, respectively, were present in the gradient fractions and no clear OMV density equilibrium point was observed (Fig 1E and 1I). Mass spectrometric analysis confirmed that these protein contaminants were rabbit and bovine serum albumin, originating from the respective growth media. As rabbit and bovine sera are essential media components for the growth of these species we removed the respective albumins from the sera by ultrafiltration through a 10 kDa molecular weight cut-off membrane and then used the filtrate as the medium supplement. A comparison of the growth of *T*. *denticola* or *T*. *forsythia* growing in media with either unfiltered or filtered sera showed no difference in growth curves (S2 Fig). Fig 1F and 1J show the albumin contaminant was removed from both OMV preparations using this technique. To reach the equilibrium density point *T*. *denticola* and *T*. *forsythia* OMVs required a 48 hour centrifugation step (150,000 x g) and gradient fractions 2, 3 and 4 (20, 25 and 30% w/v iodixanol/HEPES) and gradient fractions 3, 4 and 5 (25, 30 and 35%w/v iodixanol/HEPES) respectively, were found to contain purified OMVs (Fig 1F and 1J). *T*. *denticola* OMVs were found to be polyhedral/spherical in shape with a size distribution of 30–120 nm as determined by Cryo-TEM (Fig 1G) and a fully hydrated size range of 18–247 nm with a modal diameter of 75 nm as determined by DLS (Fig 1H). *T*. *forsythia* OMVs were found to be elliptical/spherical and to possess an EDSL surrounding the single lipid-bilayer membrane, and to have a size distribution of 80–250 nm as determined by Cryo-TEM (Fig 1K) and a fully hydrated size range of 59–397 nm with a modal diameter of 158 nm as determined by DLS (Fig 1L).

The presence of the known *P*. *gingivalis* virulence-associated activities (haemagglutination and gingipain Arg- and Lys-specific proteolytic activity) were confirmed in the *P*. *gingivalis* OMV preparations (S3 Fig and S1 Table). Neither *T*. *denticola* nor *T*. *forsythia* OMVs were able to agglutinate human or sheep RBCs nor did they exhibit significant Arg- or Lys-specific proteolytic activity (S3 Fig and S1 Table).

### Enumeration and Compositional Analysis of OMVs from the Three Periodontal Pathogens

**Fig 2:**
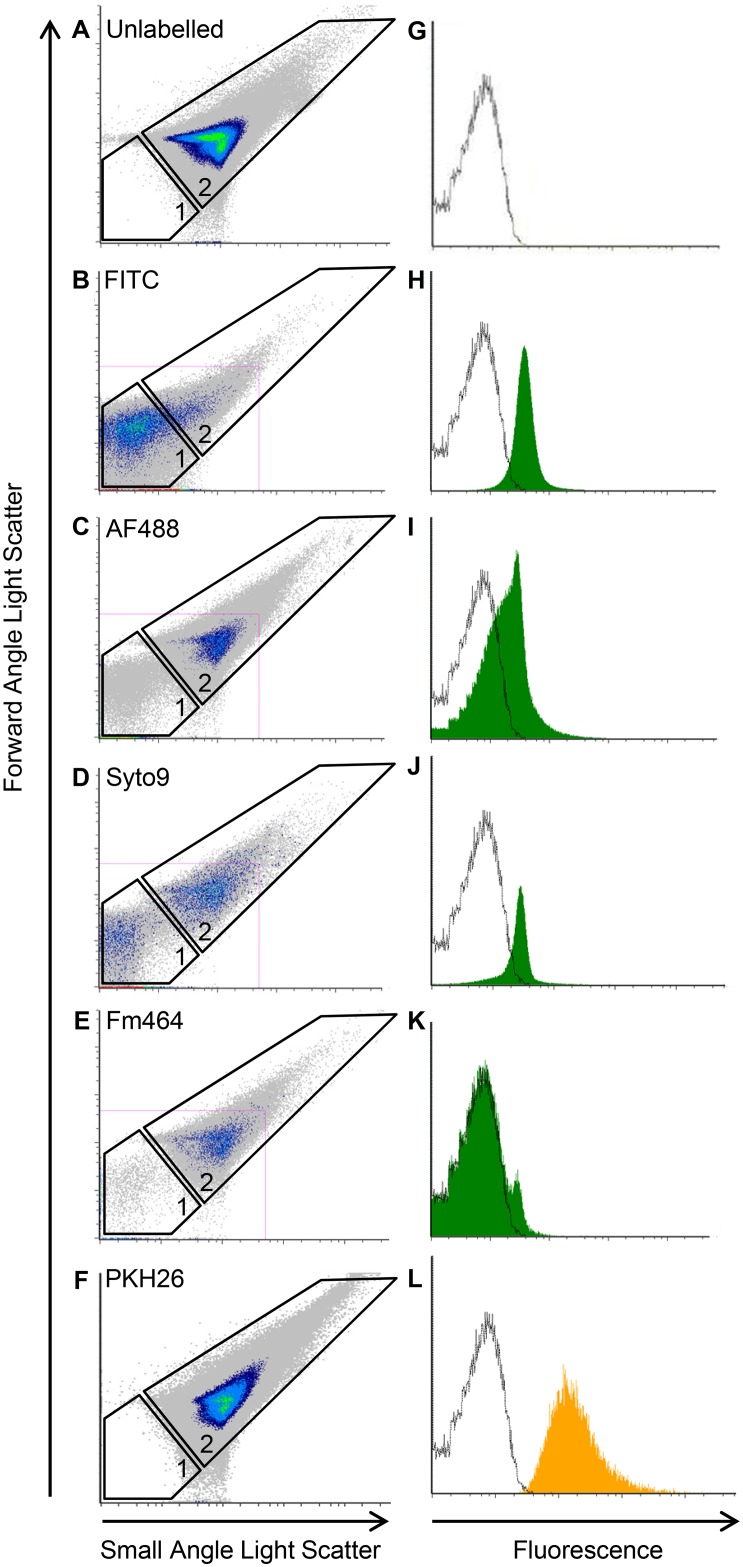
Enumeration of OMVs by high resolution flow cytometry. *P*. *gingivalis* purified OMVs unlabeled (A G) or labelled with either FITC (B H), AF488 (C I), Syto9 (D J), Fm464 (E K) or PHK-26 (F L) were analysed using an Apogee A50-Micro Flow Cytometer. Dye suitability was observed as determined by Forward/Small Angle Light Scatter (F/S-ALS) (A-F) and fluorescence (G-L).

OMVs were fluorescently labelled to enhance discrimination from background noise during flow cytometry and to provide a fluorescence trigger for enumeration. Several protein (FITC, AF488), DNA (Syto9) and lipid (Fm464, PKH-26 or PKH67) fluorescent dyes were used to label OMVs and the forward/side scatter profiles as well as fluorescence were compared with that of unlabelled OMVs (Fig 2). PKH dyes were the only dye that did not alter the OMV forward/side scatter profile, whereas FITC, AF488 and Syto9 resulted in an increase in OMV debris (Gate 1, Fig 2A–2F). Further, PKH dyes were found to provide a sufficient fluorescent shift to reliably discriminate OMVs from background noise (Fig 2G–2L). Based on these findings and the critical steps outlined in the methods OMVs from *P*. *gingivalis*, *T*. *denticola* and *T*. *forsythia* were enumerated by flow cytometry using PKH-67, FITC-labelled 110 nm flow beads in 0.1% v/v Tween 20 buffer (Table 1).

**Table 1:** Compositional analysis of OMVs from *P*. *gingivalis*, *T*. *denticola* and *T*. *forsythia*.

Parameter^a^	*P*. *gingivalis*	*T*. *denticola*	*T*. *forsythia*
OMVs/mL culture^b^	4.98 ± 0.77 x 10^12^	5.54 ± 1.47 x 10^11^	2.00 ± 0.65 x 10^12^
size range^c^	37–315 nm (121 nm)	18–247 nm (75 nm)	59–397 nm (158 nm)
OMVs/10^6^ nm^2^ cell surface area^d^	936 ± 137	158 ± 37	437 ± 90
Protein Content^e^ ng/10^8^ OMVs	38.8 ± 4.59	17.3 ± 2.47	45.4 ± 20.5
LPS/LOS Content^f^ ng/10^8^ OMVs	17.5 ± 5.21	0.11 ± 0.04	12.9 ± 0.64
Lipo-protein/peptide Content^g^ ng/10^8^ OMVs	16.2 ± 3.39	7.35 ± 0.39	14.8 ± 0.23
Peptidoglycan Content^h^ ng/10^8^ OMVs	2.97 ± 0.48	1.60 ± 0.35	5.21 ± 1.77
DNA Content^i^ pg/10^8^ OMVs	11.7 ± 3.28	7.44 ± 5.86	32.6 ± 6.74
RNA Content^j^ pg/10^8^ OMVs	5.11 ± 1.00	1.12 ± 0.50	4.42 ± 1.78

^a^ All data are presented as mean ± standard deviation of 5 biological replicates.

^b^ OMVs labelled with green fluorescent dye PHK-67 were counted using an Apogee A50-Micro Flow Cytometer in samples taken from late exponential cultures of each bacterium.

^c^ Size range of OMVs was determined using Dynamic Light Scattering on a Malvern high performance particle sizer. Modal diameter in parenthesis.

^d^ Total cell surface area was calculated using whole bacterial cell counts per mL of culture determined with a Cell Lab Quanta SC Flow Cytometer and the cellular dimensions for each species [34]

^e^ Protein concentration determined by Qubit assay.

^f^ Biologically active LPS was measured using HEK-Blue TLR4 Cells and a standard curve of *E*. *coli* LPS.

^g^ Biologically active lipoproteins were measured using HEK-Blue TLR2 Cells and a standard curve of Pam3CSK4.

^h^ Peptidoglycan was determined using a Wako SLP reagent set.

^i^ DNA was measured using a Qubit dsDNA HS assay kit.

^j^ RNA was measured using a Qubit RNA assay kit.

The amount of protein per purified OMV preparation was found to be significantly lower for *T*. *denticola* compared with that of the other pathogens (Table 1). At the point of harvesting (late exponential growth phase) *P*. *gingivalis* produced the highest number of OMVs per ml of culture and per cell surface area followed by *T*. *forsythia* and then *T*. *denticola* (Table 1).

### *P*. *gingivalis*, *T*. *denticola* and *T*. *forsythia* OMVs Differentially Activate TLR and NOD Pattern Recognition Receptors

**Fig 3:**
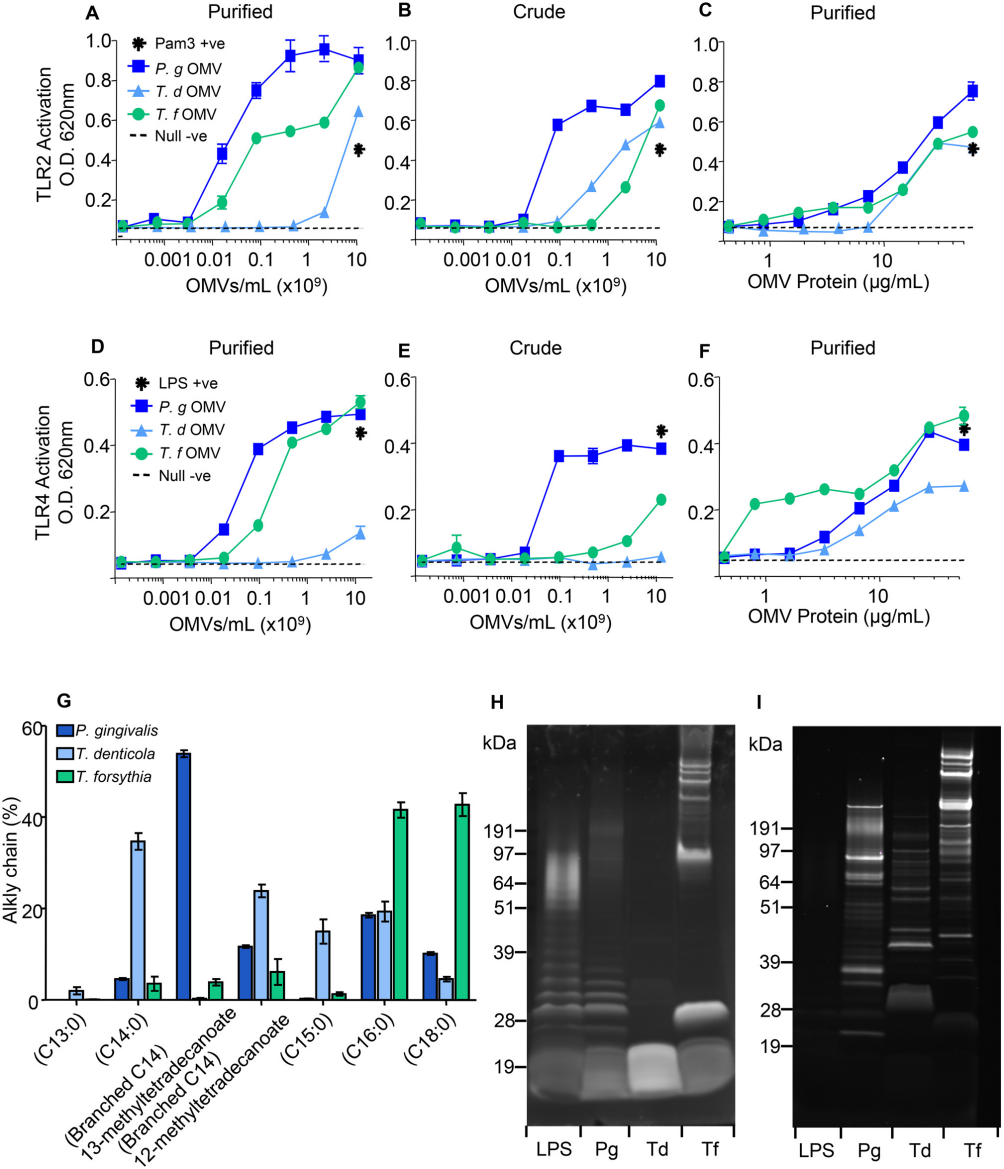
TLR2 and TLR4 Activation by *P*. *gingivalis*, *T*. *denticola* and *T*. *forsythia* OMVs. HEK-Blue TLR2 (A, B, C) and TLR4 (D, E, F) Cell lines were incubated with 20μL of either OMVs or ligands Pam3CSK4 (10 μg/mL in 5 fold dilutions) and LPS-EB (10 μg/mL in 10 fold dilutions) respectively. Alkaline phosphatase activity was determined after 20 hours incubation at 620 nm on a spectrophotometer. Crude OMV (B, E) and purified OMV preparations standardised by OMV count (A, D) and general protein (C, F) were used to ascertain the effects of purification and standardisation methods on OMV immunogenicity. Lipoproteins were observed by GC-MS fatty acid analysis to determine the % fatty acid chain lengths present (G). OMVs were subjected to SDS-PAGE and lipopolysaccharide and lipooligosaccharide detected by Pro-Q Emerald 300 LPS Gel Stain (H). Glycoproteins were identified using SyproRuby protein stain (I). Purified *P*. *gingivalis* LPS was used as a positive control. Molecular mass markers (Novex SeeBlue Plus2 Prestained Standard) are indicated in kDa next to each sample.

To investigate PRR activation by crude and purified OMVs from the three pathogens we initially compared the activation of TLR2 and TLR4 expressing HEK-Blue cells, standardised by vesicle counts or protein concentration. Fig 3A shows that purified OMVs from *P*. *gingivalis* induced a strong response in TLR2-HEK-Blue cells, with *T*. *forsythia* OMVs stimulating a slightly weaker response followed by *T*. *denticola* OMVs. Crude OMV preparations from both *P*. *gingivalis* and *T*. *forsythia* were significantly (p < 0.05) less stimulatory than purified OMVs, whereas crude *T*. *denticola* OMVs were slightly (p < 0.05) more stimulatory (Fig 3B). However, when standardised using protein concentration the stimulation of TLR2 by the different OMVs was not as well differentiated. *P*. *gingivalis* and *T*. *forsythia* OMVs were significantly (p < 0.05) less stimulatory than the same OMVs standardised by count. *T*. *denticola* OMVs were significantly (p < 0.05) more stimulatory when standardized by protein instead of counts (Fig 3C).

TLR4 expressing HEK-cells were found to be strongly activated by purified OMVs from *P*. *gingivalis*, with *T*. *forsythia* slightly less and only weakly activated by OMVs from *T*. *denticola* (Fig 3D). Crude OMVs induced significantly (p < 0.05) lower responses in TLR4 expressing cells for all of the OMVs tested (Fig 3E). Again, standardising against protein concentration resulted in the TLR4 responses being less differentiated. For *P*. *gingivalis* OMVs they were significantly (p < 0.05) less stimulatory than OMVs standardized by counts, whereas *T*. *forsythia* and *T*. *denticola* OMVs were significantly (p < 0.05) more stimulatory (Fig 3F).

The activating ligands of TLR2 are C14, C16 or C18 alkyl chains of lipopeptide/proteins with stimulation propensity being C18 > C16 > C14. Fatty acid methyl ester (FAME’s) analysis of the OMVs showed that *P*. *gingivalis* OMVs contained predominately a branched C14 alkyl chain (13-methyl-tetradecanoate, 58% of the total fatty acids) with another C14 branched (12-methyl-tetradecanoate) and linear C16 and C18 alkyl chains representing the other major alkyl chains present (Fig 3G). *T*. *denticola* OMVs possessed four major alkyl chains; saturated linear C14, C15 and C16 and a branched C15 alkyl chain (Fig 3G). *T*. *forsythia* OMVs contained predominately two major alkyl chains of similar abundance; C16 and C18 linear alkyl chains at 42% and 43% respectively as well as small amounts of branched and unbranched C14/C15 alkyl chains (Fig 3G). Biologically active lipo-protein/peptide concentrations (ng per 10^8^ OMVs) revealed *P*. *gingivalis* OMVs contained the most lipo-protein/peptide, followed by *T*. *forsythia* and then *T*. *denticola* (Table 1).

The activating ligands for TLR4 include the lipid anchors of LPS and LOS. The presence of LPS in OMVs was determined by Pro-Q Emerald LPS Staining. Traditional smooth LPS was identified in the *P*. *gingivalis* OMV sample, which Pro-Q Emerald stained as the typical laddered banding pattern (Fig 3H). LOS, observed as a low molecular weight smeared band with no laddered banding was identified in both *T*. *denticola* and *T*. *forsythia* OMV samples (Fig 3H). Additional high molecular weight bands in the *T*. *forsythia* OMV sample appeared to be glycoproteins reacting with the Pro-Q Emerald stain as these bands counter stained with the protein stain SyproRuby (Fig 3I). Biologically active LPS/LOS concentrations (ng per 10^8^ OMVs) revealed that *P*. *gingivalis* OMVs contained the most LPS/LOS, followed by *T*. *forsythia* and then *T*. *denticola* (Table 1).

**Fig 4:**
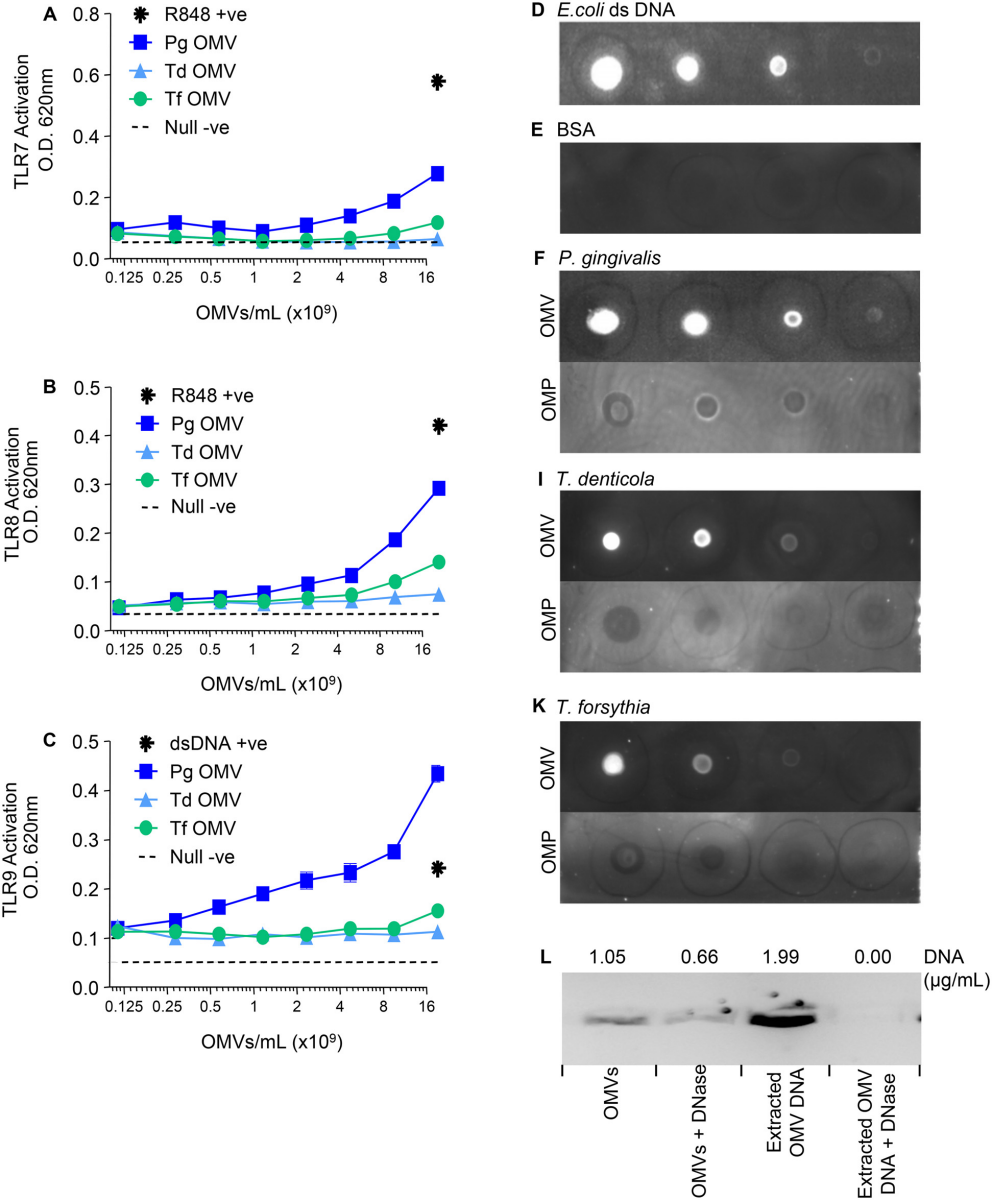
TLR7, TLR8 and TLR9 Activation by *P*. *gingivalis*, *T*. *denticola and T*. *forsythia* OMVs. HEK-Blue TLR7 (A), TLR8 (B) and TLR9 (C) Cell lines were challenged with 20μL of either purified OMVs or ligands R848 (10 μg/mL in 2 fold dilutions) and dsDNA (100 μg/mL in 2 fold dilutions) respectively. Alkaline phosphatase secretion was determined after 20 hours incubation at 620nm on a spectrophotometer. Monoclonal antibody αdsDNA MAB030 was used to detect DNA in purified OMV preparations and Triton X-114 extracted Outer Membrane Protein (OMP) preparations, used at 0.5 mg/mL protein and 1.5 mg/mL protein respectively, for *P*. *gingivalis* (F), *T*. *denticola* (I) and *T*. *forsythia* (K). OMV preparations were spotted on a nitrocellulose Immuno-Blot PVDF Membrane for Protein Blotting in three 10-fold dilutions. *E*. *coli* double stranded DNA was used as a positive control at 50 ng/mL in three 10-fold dilutions (D). Bovine Serum Albumin was used as a negative control at 0.5 mg/mL in three 10-fold dilutions (E). *P*. *gingivalis* whole OMVs and extracted OMV DNA were treated with DNase and DNA concentrations determined using Qubit Assay Kits and SYBR Safe DNA gel stain (L).

To investigate whether *P*. *gingivalis*, *T*. *denticola* and/or *T*. *forsythia* OMVs stimulate TLRs that recognise RNA (TLR7 and TLR8) and unmethylated DNA (TLR9), purified OMVs from each bacterium were incubated with TLR7, TLR8 and TLR9 expressing HEK-Blue cells. Fig 4A–4C shows that HEK expressing TLR7, TLR8 and TLR9 cells responded moderately/strongly to *P*. *gingivalis* OMVs, weakly to *T*. *forsythia* OMVs and not at all to *T*. *denticola* OMVs. All three purified OMVs were found to be positive for DNA and RNA with the *T*. *denticola* vesicles containing the lowest amounts (Table 1). We further confirmed the presence of OMV-associated DNA using DNA-specific monoclonal antibody mAb (α-dsDNA MAB030). The specificity of the antibody was confirmed using extracted *E*. *coli* dsDNA and DNA free BSA (Fig 4D and 4E). The DNA specific antibody recognised all of the OMV preparations but did not bind to the corresponding Triton X-114 extracted outer membrane protein preparations (Fig 4F–4K). Finally, purified *P*. *gingivalis* OMVs and extracted OMV-associated DNA were treated with DNase. OMV-extracted DNA was completed digested by DNase as indicated by an absence of DNA stain and no detection by the Qubit DNA assay. However, whole OMVs treated with DNase for the same period were positive for DNA indicating that a proportion of OMV-associated DNA was inaccessible to DNase (Fig 4L).

**Fig 5:**
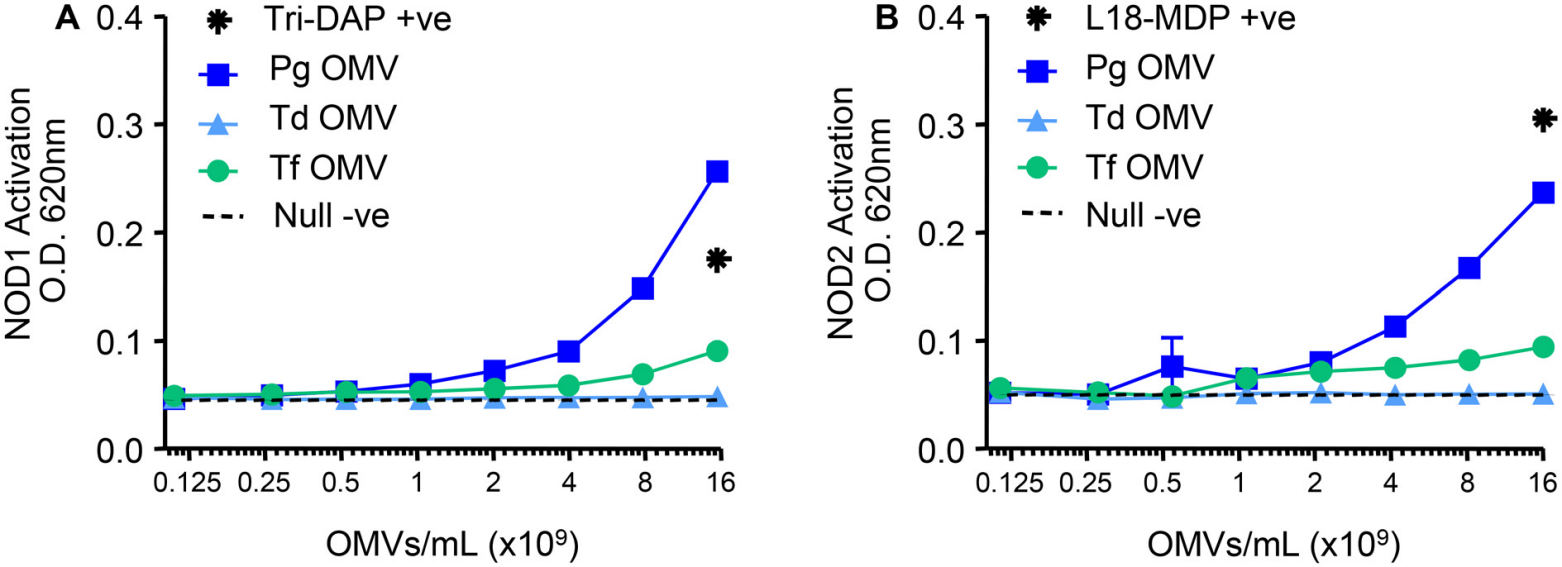
NOD1 and NOD2 Activation by *P*. *gingivalis*, *T*. *denticola and T*. *forsythia* OMVs. HEK-Blue NOD1 (A) and NOD2 (B) Cell lines were challenged with 20 μL of either purified OMVs or ligands Tri-DAP (150 μg/mL in 2 fold dilutions) and L18-MDP (50 μg/mL in 2 fold dilutions), respectively. Alkaline phosphatase secretion was determined after 20 hours incubation at 620 nm on a spectrophotometer.

Peptidoglycan was detected in *P*. *gingivalis*, *T*. *denticola* and *T*. *forsythia* OMVs using a silkworm larvae plasma (SLP) assay (Table 1). To determine whether *P*. *gingivalis*, *T*. *denticola* and/or *T*. *forsythia* OMVs stimulate NLRs that recognise peptidoglycan, purified OMVs from each bacterium were incubated with NOD1 and NOD2 expressing HEK cells. Fig 5A and 5B shows that HEK cells expressing NOD1 and NOD2 responded moderately/strongly to *P*. *gingivalis* OMVs, weakly to *T*. *forsythia* OMVs, and not at all to *T*. *denticola* OMVs.

## Discussion

Outer membrane vesicles are being increasingly recognised as major virulence determinants for Gram-negative bacteria. Composition and media contamination of OMVs prepared under different conditions or from different bacterial species can vary considerably [22, 23]. The results of the current study show that immunological effects can be better differentiated when highly purified OMVs are used and standardized using vesicle number. To standardize OMVs on vesicle number we developed an enumeration method using an Apogee A50-Micro Flow Cytometer and found that different fluorescent dyes had effects on OMV stability. Labelling OMVs with protein or DNA dyes resulted in an increase in particle debris, indicating that labelled OMVs were less stable than unlabelled OMVs. The lipid dye Fm464 also increased OMV debris slightly whereas the lipid dyes PKH26 and PKH67 had no effect on OMV stability and also provided the largest fluorescent shift from background. Similar to our findings with bacterial OMVs, van der Vlist et al. [27] found that another PKH lipid dye, PKH67, gave the brightest signal when used for the enumeration of mammalian exosomes. These results suggest that lipid dyes, in particular PKH dyes, are preferable to protein and DNA labelling dyes for OMV enumeration. Our initial findings that setting cytometer flow and event rates using μm diameter flow beads significantly underestimated OMVs/mL, was in agreement with Wieser et al [29]. who used a formula based on a set OMV diameter (100 nm) and the size/volume differences between the flow bead and set OMV size to convert OMV event rate to counts. Using flow beads (110 nm) that are within the size distribution of the OMV samples to set the flow and event rates, labelling OMVs so that they fluoresce in the same channel as the flow bead and minimising OMV aggregation, the protocol described here provides a count of the OMV sample which takes into account the size distribution.

The optimised OMV purification method described here produced highly purified single lipid-bilayer vesicles that were shown to be immunologically active. OMVs were generally spherical in shape with slight variations including a tendency to polyhedral and elliptical shapes for *T*. *denticola* and *T*. *forsythia* OMVs, respectively. Size distribution profiles observed were largely in agreement with previous electron microscopy studies. Both *P*. *gingivalis* and *T*. *forsythia* OMVs were observed to have 20 nm thick electron dense surface layers which have been described as contributing virulence factors [13, 19].

OMV enumeration revealed that for all three periodontal pathogens vesicles were produced during late exponential growth phase. Several theories exist on the mechanism of vesiculation [1, 35, 36] but production is thought to increase upon restricted growth conditions and/or environmental stress [36, 37]. *P*. *gingivalis* OMV gravimetric yields are known to increase when the bacterium is grown in haemin-restricted growth conditions [26]. Bacterial cultures in this study were harvested at late exponential growth where they would be just starting to experience environmental and nutritional stress. Under these conditions all three pathogens produced significant quantities of OMVs. For *P*. *gingivalis* the ratio of cells to OMVs was found to be approximately 1:2,000. Several studies have shown that the level of *P*. *gingivalis* in subgingival plaque is associated with disease severity and that a threshold of around 10^6^ *P*. *gingivalis* cells per site is required for disease progression [38–40]. In our study we used a maximal 1 x 10^10^ OMVs/mL equating to 5 x 10^6^ *P*. *gingivalis*/mL so as to mimic periodontitis conditions.

In our initial studies on OMV stimulation of PRRs, we investigated whether there was a difference between PRR activation by OMVs standardised by counts or protein as well as the effect of the additional Optiprep purification. Optiprep purified *P*. *gingivalis* and *T*. *forsythia* OMVs induced a stronger TLR2 and TLR4 response when compared with the equivalent crude preparations. Unknown cell or media derived PRR dampening substances may be associated with the crude OMV preparations which were removed during the Optiprep purification step. However, *T*. *denticola* crude OMV preparations were found to be more potent TLR2 activators. This may be attributable to the presence of lipoprotein-rich cell debris pelleting with OMVs in the crude preparation. Standardization of OMVs based on protein content rather than on vesicle number reduced the ability to differentiate OMV PRR activation between the three pathogens. These findings highlight the importance of using highly purified OMV preparations standardized by vesicle number when performing immune cell activation assays.

Purified OMVs from all three periodontal pathogens possessed ligands capable of either strong/moderate (*P*. *gingivalis*), moderate (*T*. *forsythia*) or weak (*T*. *denticola*) activation of TLR2 and TLR4. In addition *P*. *gingivalis* and *T*. *forsythia* OMV preparations were capable of moderate and weak activation respectively, of TLR7, TLR8, TLR9, NOD1 and NOD2. While each of these oral pathogens has been associated with periodontitis, *P*. *gingivalis* is regarded as the principal or keystone pathogen while *T*. *denticola* and *T*. *forsythia* are regarded as accessory pathogens playing a synergistic or supporting role [34, 41–44]. The results of the current study indicate that *P*. *gingivalis* OMVs exhibited greater stimulatory activity of PRRs when compared with OMVs from *T*. *denticola* and *T*. *forsythia*. *P*. *gingivalis* also produced greater numbers of OMVs when compared with the other two pathogens. Hence taken together the results are consistent with the dominant role of *P*. *gingivalis* in periodontitis. PRR activation is known to play a significant role in the many aspects of the chronic inflammation associated with periodontal tissue destruction. TLR2, TLR4 and TLR9 expression is increased in severely inflamed gingival tissue, particularly the connective tissue subjacent to the periodontal pocket epithelium and the subgingival plaque biofilm [45]. TLR2 activation is critical for human gingival epithelial cells [46] and human macrophages [47] to recognise and respond to *T*. *denticola* and both TLR9 and NOD2 have been reported to contribute to *P*. *gingivalis*-induced bone loss in an experimental model of periodontitis [48, 49]. Even though *P*. *gingivalis* and *T*. *forsythia* DNA induce pro-inflammatory cytokine production through TLR9 signalling [50], TLR2 and TLR4 have been implicated as the critical receptors to control the pro-inflammatory cytokines IL-1β, IL-6, IL-8 and TNFα induced by the pathogens [51]. Gingival tissues of chronic periodontitis patients exhibit significantly higher levels of pro-inflammatory cytokines IL-1, IL-6, IL-8 and TNFα than those of periodontally healthy patients [52, 53]. The inflammatory effects of these cytokines have been extensively reviewed and include activation of neutrophils, T and B lymphocytes, macrophages, Natural Killer cells and osteoclasts to promote connective tissue destruction and alveolar bone resorption, the clinical hallmarks of chronic periodontitis [54–56].

*P*. *gingivalis*, *T*. *denticola* and *T*. *forsythia* OMV composition was investigated to correlate with the TLR and NLR activation seen using HEK-Blue cell lines. TLR2 recognizes a diverse range of bacterial products, including peptidoglycans, lipoteichoic acid and lipopeptides [57] and OMV preparations from all three pathogens induced TLR2 responses and contained at least one of these ligands. The immunogenicity of lipopeptides is thought to be dependent on the length of their alkyl chains; chain lengths C18, C16 and possibly C14 induce stronger host responses than C12 and C8 [58]. Lipid analysis of OMVs revealed all possessed alkyl chain lengths of C14, C16 and C18 in varying concentrations. *T*. *forsythia* OMVs comprised predominately alkyl chain lengths C18 and C16, which may explain their activation of TLR2. *P*. *gingivalis* OMVs exhibited the second greatest percentage of C16/C18 but the majority of alkyl chains were branched C14, which is as yet unexplored as a TLR2 stimulant. *T*. *denticola* OMVs possessed the least amount of C18 and contained more conventional saturated C14, which was consistent with it being the least TLR2 stimulatory of the three pathogens.

In this study HEK-Blue TLR4 cells were activated strongly/moderately by *P*. *gingivalis*, moderately by *T*. *forsythia* OMVs and only weakly by *T*. *denticola* OMVs and this is likely a reflection of their different LPS/LOS structures. *P*. *gingivalis* possesses the most conventional lipopolysaccharide of the three pathogens: smooth traditional LPS detected as a laddered banding pattern by Pro-Q Emerald LPS Stain. Although *P*. *gingivalis* LPS has been described as atypical the penta-acylated form of its lipid A anchor has been implicated in TLR4 activation [59]. There has been controversy as to whether *P*. *gingivalis* LPS also activates TLR2 although studies with synthetic *P*. *gingivalis* Lipid A have demonstrated only TLR4 activation [60, 61]. Furthermore, TLR2 activating lipoproteins have been identified in *P*. *gingivalis* LPS preparations that are resistant to re-extraction methods used for other Gram negative bacteria [62]. In the current study *P*. *gingivalis* OMV preparations were able to strongly activate both TLRs, particularly in comparison with *T*. *denticola* OMVs. While *T*. *denticola* and *T*. *forsythia* do not possess the traditional LPS ladder, both contained equivalent molecules which are likely to be capable of stimulating TLR4. *T*. *forsythia* possesses a rough-type LPS with various immunological activities including the induction of pro-inflammatory cytokines from human macrophages [63]. However very little information is available regarding the lipid anchor structure of this LPS. Treponemes lack genes encoding the necessary enzymes for LPS synthesis; as such *T*. *denticola* does not possess typical LPS but lipooligosaccharides (LOS) [64]. While functionally similar to traditional LPS, the membrane anchor of *T*. *denticola* LOS is more closely related to lipoteichoic acids of Gram positive bacteria than lipid A, but is still known to stimulate TLR4 [65]. The results of the current study suggest that *T*. *denticola* LOS, as present on purified OMVs, is a less potent stimulant of TLR4 than the LPS on *P*. *gingivalis* or *T*. *forsythia* OMVs.

The presence of DNA and RNA in/on OMVs has been widely debated. This current study demonstrated that pure OMVs have associated nucleic acids and activate TLR7, TLR8 and TLR9 which are known to recognise bacterial DNA and RNA. A proportion of this DNA remained deoxyribonuclease (DNAse) inaccessible, indicating that it may have been located intra-vesicularly as has been proposed for outer membrane vesicles from other bacterial species [66, 67]. Analysis of the culture fluid for each species confirmed the presence of significant amounts of extracellular DNA. The amount of extracellular DNA found associated with whole cells, OMVs and free in the culture media suggests that all three pathogens are capable of DNA secretion, a common mechanism (alongside cell lysis) for the release of DNA in bacterial cultures [68].

The presence of extracellular DNA (eDNA) in the purified OMV preparations suggests that the DNA strands may be bound to the surface of the OMVs potentially linking them in an eDNA/OMV network. It is interesting to speculate how this eDNA/OMV net could be used in nutrient capture by the pathogens residing at the surface of a polymicrobial biofilm. A gingival inflammatory exudate (crevicular fluid) containing essential nutrients (protein/peptides and heme) flows over the surface of the biofilm such that a released eDNA/OMV net into the nutrient stream may enhance survival of sessile bacteria by extending their sphere of influence in nutrient capture.

NOD1 and NOD2 are cytosolic PRRs that sense peptidoglycan derived diaminopimelic acid and muramyl dipeptide respectively [69]. Several bacterial species are known to release peptidoglycan fragments during normal cell growth including *Neisseria meningitides* [70*]*, *Neisseria gonorrhoeae* [71] and *Bordetella pertussis* [72]. These released peptidoglycan fragments can induce inflammatory cytokine production and promote host cell death. OMV-associated peptidoglycan may be a part of the internal structure of OMVs, incorporated during biogenesis or merely surface bound extracellular fragments derived from released peptidoglycan. In either case, OMVs have been identified as a medium for the delivery of peptidoglycan to cytosolic NOD1 receptors for *Helicobacter pylori*, *Pseudomonas aeruginosa*, and *Neisseria gonorrhoea* [73]. The activation of Silkworm Larvae Plasma (SLP) in this study by all purified OMV preparations from the three periodontal pathogens suggests the presence of peptidoglycan fragments. However, SLP is not specific for diaminopimelic acid or muramyl dipeptide; it is a prophenoloxidase cascade system initiated by the binding of an unknown peptidoglycan epitope. This may explain why peptidoglycan concentrations determined by SLP activation did not correlate well with HEK-Blue NOD1 and NOD2 activation; where *P*. *gingivalis* OMVs induced the greatest response, followed by *T*. *forsythia*. *T*. *denticola* OMVs did not induce activation. While SLP activation indicated the presence of peptidoglycan in *T*. *denticola* OMVs it was either in a form or location inaccessible to the extracellular NOD1 and NOD2 receptors of the HEK-Blue Cells.

In conclusion, we have developed procedures for purifying and enumerating OMVs. Purified OMVs were analysed to identify protein, fatty acids, lipopolysaccharide, peptidoglycan and nucleic acids. Using HEK-Blue cell lines stably transfected with TLR and NOD receptors we demonstrated that OMVs may trigger significant inflammatory responses via multiple signalling pathways. *P*. *gingivalis* OMV preparations induced the greatest PRR response per vesicle followed by *T*. *forsythia* OMVs and finally *T*. *denticola* OMVs. The results demonstrate that periodontal pathogen OMVs are stimulants of PRRs and therefore are likely to have a role in the chronic inflammatory pathology of periodontitis.

## Supporting Information

S1 Experimental Procedures (DOCX)

S1 Fig TEM of OMVs present in bacterial pellet. (DOCX)

S2 Fig Growth curve of *T*. *denticola* and *T*. *forsythia*. (DOCX)

S3 Fig Haemagglutination activity of *P*. *gingivalis*, *T*. *denticola* and *T*. *forsythia* OMVs. (DOCX)

S1 Table Protease activity of *P*. *gingivalis*, *T*. *denticola* and *T*. *forsythia* OMVs. (DOCX)
