# Supplementary material for: Differential Responses of Pattern Recognition Receptors to Outer Membrane Vesicles of Three Periodontal Pathogens
Source: PLoS One. 2016 Apr 1;11(4):e0151967. doi: 10.1371/journal.pone.0151967 (PMC4818014; doi:10.1371/journal.pone.0151967)
Supplement: S1 Experimental Procedures — (DOCX) [file pone.0151967.s001.docx]

**S1 Experimental Procedures**

**Bacterial Cultures and Growth Conditions**

*P. gingivalis* W50 was maintained on horse blood agar (HBA), comprised of 4% w/v Blood Agar Base No. 2 (**Thermo Fisher Scientific, SA, Australia**) supplemented with 10% v/v defibrinated horse blood (Equicell, Australia) and 1% w/v menadione (Sigma-Aldrich, NSW, Australia) in a MK3 Anaerobic Workstation (Don Whitley Scientific Limited, NSW, Australia) at 37°C with a gas composition of 5% v/v H_2_, 10% v/v CO_2_ in N_2_ (BOC Gases Australia, NSW, Australia). *P. gingivalis* W50 was cultured in volumes of 800 mL in 3.7% w/v Bacto Brain Heart Infusion media broth (BD, NSW, Australia) supplemented with 0.5% w/v hemin (Merck Millipore, Vic, Australia) and 0.1% w/v cysteine (Sigma-Aldrich, NSW, Australia). The culture medium was pH 7.4 and the bacteria were grown to late exponential phase at 37 °C under anaerobic conditions to an optical density (OD) of 1.0 at 600 nm.

*T. denticola* ATCC 35405 was cultured in in volumes of 400 mL of Oral Bacteria Growth Media (OBGM) comprised of 1.25% w/v Bacto Brain Heart Infusion media broth, 1% w/v trypic soy broth (BD, NSW, Australia), 0.75% w/v yeast extract (**Thermo Fisher Scientific, SA, Australia**), 0.2% w/v sodium chloride (VWR International, QLD, Australia), 0.2% w/v ascorbic acid (Sigma-Aldrich, NSW, Australia), 0.2% w/v D-glucose (Chem Supply, SA, Australia), 0.1% w/v pyruvic acid (Sigma-Aldrich, NSW, Australia), 0.05% w/v sodium thioglycate (Sigma-Aldrich, NSW, Australia) and 0.025% w/v asparagines (Sigma-Aldrich, NSW, Australia) supplemented with 0.2% w/v sodium bicarbonate (Chem Supply, SA, Australia), 0.2% g ammonium sulphate (Chem Supply, SA, Australia), 0.1% w/v cysteine, 0.6% w/v thiamine pyrophosphate (Sigma-Aldrich, NSW, Australia), 0.5% w/v hemin, 0.05% w/v menadione, 2.5% v/v heat inactivated Rabbit Serum (Sigma-Aldrich, NSW, Australia) filtered using a Vivaspin10kDa MWCO (GE HealthCare Life Sciences, NSW, Australia) at 8,000 x g for 1 hour at 4 ^o^C and 0.0025% v/v volatile fatty acid mix containing 0.5% v/v isobutyric acid, 0.5% v/v DL-1-methylbutyric acid, 0.5% v/v isovaleric acid and 0.5% v/v valeric acid in 0.1M potassium hydroxide. The culture medium was pH 7.4 and the bacteria were grown to late exponential phase (OD 0.3) at 37 °C under anaerobic conditions.

*T. forsythia* ATCC 43037 was cultured in volumes of 400 mL in tryptic soy broth with yeast extract and vitamin K (TSBYK), comprised of 1.85% w/v Bacto Brain Heart Infusion media broth, 1.5% w/v tryptic soy broth and 1.0% w/v yeast extract supplemented with 0.1% w/v cysteine, 0.1% N-Acetylmuranic acid (Sigma-Aldrich, NSW, Australia), 0.5% w/v hemin, 0.05% menadione and 5% v/v heat inactivated Fetal Calf Serum filtered using a Vivaspin10kDa MWCO (GE HealthCare Life Sciences, NSW, Australia) at 8,000 x g for 1 hour at 4 ^o^C . The culture medium was pH 7.4 and the bacteria were grown to late exponential phase (OD 1.0) at 37 °C under anaerobic conditions.

**Haemagglutination Assay**

OMV preparations were diluted across round bottomed 96 well plates (Interpath Services, VIC, Australia) in PBS by 2-fold serial dilutions such that the final volume of each well was 50μL. 50μL of 0.5% v/v washed RBCs in PBS was added to each well and haemagglutination observed after an incubation of 4 hours at room temperature. The titration end point was determined as the protein concentration at which OMVs no longer induced the complete haemagglutination of RBCs.

**Arg-x-specific and Lys-x-specific Proteinase Assays**

Benzoyl-L-Arg-p-nitroanilide (Bz-L-Arg-pNA) (Sigma-Aldrich NSW, Australia) and N-p-Tosyl-Gly-Pro-Lys 4-nitroanilide acetate salt (z-L-Lys-p-NA) (Sigma-Aldrich NSW, Australia) were used to assay for Arg- and Lys- proteolytic activity, respectively. 2 mM Bz-L-Arg-pNA or 2 mM z-L-Lys-p-NA were suspended in 3 ml isopropan-2-ol with 7 ml of enzyme buffer (400 mM Tris-HCl, 100 mM NaCl and 20 mM L-cysteine; pH 8.0). *T. denticola*, *T. forsythia* and *P. gingivalis* OMV preparations or purified RgpA-Kgp proteinase-adhesin complexes of *P. gingivalis* were made up to the desired concentration in TC150 buffer (50mM Tris-HCl, 150 mM NaCl, 5mM CaCl_2_; pH 8.0) supplemented with 10mM L-cysteine (pH 8.0) to 100µl and incubated for 10 min at 37°C in a 96-well microtitre plate. 100µl of Bz-L-Arg-pNA or z-L-Lys-p-NA were added and proteinase activity was measured every 4s for 30min at 37°C using a Wallac, Victor 3 microtitre plate reader (PerkinElmer, MA, USA) at a wavelength of 405nm. Proteolytic activity is expressed as units (micromoles of substrate converted per minute) at 37°C.

**Lipopolysaccharide Assay**

Pro-Q Emerald 300 LPS Gel Stain (Life Technologies, NSW, Australia) was utilised to identify LPS in OMV preparations. Briefly 30uL of OMV sample was denatured with 30uL Tricine SDS Sample Buffer and separated by SDS-PAGE using NuPAGE^®^ Novex 10-20% Tricine gels with Tricine SDS Running Buffer (Life Technologies, NSW Australia). The gel was treated and stained with Pro-Q Emerald 300 Staining Solution according to the manufacturer’s instructions. Additional SyproRuby (Life Technologies, NSW Australia) protein staining was performed on the Tricine gel to detect the presence of glycoproteins cross-reacting with Pro-Q Emerald 300 LPS Gel Stain.

**Fatty Acid Detection Assays**

Extraction of OMVs for GC-MS fatty acid analysis was performed using a modified method previously reported by Llorente et al. ([Llorente A, 2013](#_ENREF_1)). A 150 µL aliquot of vesicles was transferred into an Eppendorf tube (2mL). Methanol (500 µL) was added and the sample was vortexed (60 sec) and centrifuged at 13,000 rpm for 15 min at room temperature (23°C). Chloroform (1000 µL) was added and the mixture was vortexed (60 sec), centrifuged at 13,000 rpm for 15 min at room temperature (23°C) and sonicated (60 sec) to re-suspend the vesicle pellet. The mixture was subsequently centrifuged at 13,000 rpm for 15 min at room temperature (23°C). The resultant supernatant was transferred into a new Eppendorf tube upon the addition of 0.1M HCl (600 µL) and chloroform (750 µL) to facilitate phase separation. The mixture was then centrifuged at 13,000 rpm for 5 min at room temperature (23°C). The upper polar phase was removed and the bottom layer was then dried down using a Rotational Vacuum Concentrator (CHRIST, Model: RVC-2-33) in the corresponding Eppendorf tube. The dried vesicle extract was then resuspended in a 1:1 mixture of butanol:methanol containing 10mM ammonium carbonate. A 60 µL aliquot was transferred into glass insert and dried *in vacuo* for GC-MS fatty acid analysis. Extracted vesicle samples were placed in a snaplock bag with silica gel prior to derivatisation for GC-MS analysis.

The dried sample was reconstituted in 25 µL of (2:1) chloroform:methanol. Meth-Prep™ II (5 µL; Grace Davison Discovery) was added and then immediately crimped with a magnetic closure. Samples were then incubated for 30 min at a temperature of 37°C using an agitator speed of 500 rpm. Each derivatised sample was allowed to rest for 10 min prior to injection.

Samples (1 μL) were injected in splitless mode into a GC-MS system comprised of a Gerstel 2.5.2 autosampler, a 7890A Agilent gas chromatograph and a 5975C Agilent quadrupole MS (Agilent, Santa Clara, USA). The MS was adjusted according to the manufacturer’s recommendations using *tris*-(perfluorobutyl)-amine (CF43). The GC was performed on a 30 m VF-5MS column with 0.2 μm film thickness and a 10 m Integra guard column (J & W, Agilent). The injection temperature was set at 250°C, the MS transfer line at 280°C, the ion source adjusted to 250°C and the quadrupole at 150ºC. Helium was used as the carrier gas at a flow rate of 1.0 mL min^-1^. For the fatty acid analysis, the following temperature program was used; start at injection 50°C, hold for 1 min, followed by a 15°C min^-1^ oven temperature ramp to 230°C; hold for 3 min, followed by a 10°C min^-1^ ramp to 300°C. Mass spectra were recorded at 2 scans s^-1^ with an *m/z* 50-600 scanning range. Both chromatograms and mass spectra were evaluated using the Agilent MassHunter Workstation Software, Quantitative Analysis, Version B.05.00/Build 5.0.291.0 for GCMS. The retention times and mass spectra (unique qualifier ions) were identified and compared directly using a commercially available fatty acid methyl ester mix (Supelco-37 Component FAME Mix, 47885-U, Sigma–Aldrich, Castle Hill, NSW, Australia). All fatty acid methyl esters identified were quantified using prepared calibration curves from the stock Supelco-37 Component FAME Mix in the linear range from 0 to 200 µM as described in Olmstead et al. ([Olmstead ILD, 2013](#_ENREF_2))

**LPS/LOS and Lipoprotein/peptide quantification**

HEK-Blue cells were treated with OMV samples and controls as outlined in the S1 Experimental Procedures section: Toll-like Receptor (TLR) Activation Assay. Biologically active LPS/LOS and lipoprotein/peptides were quantified at maximum activation using HEK-Blue TLR4 Cells with a standard curve of *E. coli* LPS and HEK-Blue TLR2 Cells with a standard curve of Pam3CSK4.

**Nucleic Acid Detection Assays**

Nucleic acids were identified in OMV preparations using a Qubit dsDNA HS Assay Kit (Life Technologies NSW Australia) and Qubit RNA Assay Kit (Life Technologies NSW Australia) according to the manufacturer’s instructions. Nucleic acids were also identified in OMV preparations using SYBR Safe DNA gel stain (Life Technologies, NSW, Australia) according to the manufacturer’s instructions. Briefly, OMV preparations were diluted across flat bottomed 96 well plates (Interpath Services, VIC, Australia) in PBS by 2-fold serial dilutions such that the final volume of each well was 50 μL. 50 μL of 1/5000 SYBR Safe DNA gel stain in PBS was added to each well and fluorescence determined at 485 nm/535 nm on a spectrophotometer.

Nucleic acids were extracted from OMV samples using an SDS/TE Buffer protocol. Briefly, 100 μL of purified OMVs was treated with 100 μL of 20% SDS (w/v), 3 mL of TE Buffer (10 mM Tris, 1 mM EDTA, pH 8.0) and 1 mL of saturated NaCl solution. After 2 washes in ethanol (100% then 70% EtOH) at 20,000 x g for 30 min at 4 °C (using a JA-12 rotor installed in an Avanti J-25I Centrifuge, Beckman Coulter, NSW, Australia), the resulting pellet was air dried and resuspended in 200 μL PBS. Both whole vesicles and extracted nucleic acid were treated with 50 mg/mL DNase, at a 2:1 ratio for 1 hour at 37^o^C before additional DNA and RNA quantification using Qubit Assay Kits and SYBR Safe DNA gel stain as previously described.

For DNA detection using αdsDNA MAB030 antibody (Millipore), 2 μL of extracted nucleic acid was spotted directly onto a nitrocellulose Immun-Blot PVDF Membrane for Protein Blotting (BioRad) in three 10-fold dilutions. Purified BSA (BioLabs) was used at a starting concentration of 0.5 mg/mL in three 10-fold dilutions. *E.coli* dsDNA (Life Technologies, NSW, Australia) was used at a starting concentration of 50 ng/mL in three 10-fold dilutions. The nitrocellulose membrane was treated with UV light for 1 minute and non-specific sites blocked with 5% w/v skim milk in TBS-T (0.05% v/v Tween20, 20 mM Tris-HCl, 150 mM NaCl) for 1 hour at room temperature. The nitrocellulose membrane was incubated with primary antibody αdsDNA MAB030 (Millipere) at 1: 10,000 in 0.1% w/v skim/TBS-T for 30 minutes at room temperature. The membrane was washed three times in TBS-T and incubated with secondary antibody Goat-Anti Mouse IgG-2a at 1: 2,000 in TBS-T for 30 minutes at room temperature. The membrane was washed in TBS-T and incubated with tertiary antibody Swine Anti-Goat IgG – HRP at 1: 4,000 in TBS-T for 30 minutes at room temperature. The membrane was washed three times with TBS-T (15 minutes x 1, 5 minutes x 2) and once with TBS (20 mM Tris-HCl, 150 mM NaCl) for 5 minutes at room temperature. The membrane was incubated with Enzyme Substrate Immobilon Western Substrate – HRP Substrate (Immobilon) for 1 minute to visualise antibody binding.

**Peptidoglycan Detection Assay**

Peptidoglycan was detected in OMVs using an SLP Reagent Set (Wako) according to the manufacturer’s instructions. A standard curve was produced using controls of a known peptidoglycan concentration (isolated from *Bacillus subtilis*).

**Toll-like Receptor (TLR) Activation Assay**

HEK-Blue cell lines Null, TLR2, TLR4, TLR7, TLR8, TLR9, NOD1 and NOD2 (Invivogen, USA) were grown at 37 ^o^C in an anaerobic chamber in complete Dulbecco’s Modified Eagles Medium (DMEM, 10% v/v Fetal Calf Serum, 3.5% v/v Glucose, 1% v/v Pen/Strep, 1% v/v L-Glut) with selective antibiotics specific for each cell line; Null (100 μg/mL Zeocin, 100 μg/mL Normocin), TLR7 and TLR9 (100 μg/mL Zeocin, 100 μg/mL Normocin, 10 μg/mL Blasticidin), TLR8, NOD1 and NOD2 (100 μg/mL Zeocin, 100 μg/mL Normocin, 30 μg/mL Blasticidin), TLR2 and TLR4 (100 μg/mL Zeocin, 100μg/mL Normocin, 30 μg/mL Blasticidin, 200 μg/mL Hygromycin). Cells were removed from culture flasks (Corning, VIC, Australia) by gentle tapping and counted using a Z1 Coulter Particle Counter (Beckman Coulter, NSW, Australia). A flat bottomed 96-well plate (Interpath Services, VIC, Australia) was seeded with 2 x 10^4^ cells per well in 200 μL of complete DMEM and incubated for 24 hours at 37 °C under anaerobic conditions. Supernatant was aspirated and 200 μL of fresh, warmed DMEM added. TLR2 and TLR4 cell lines were challenged with 20 μL of OMVs in PBS starting at 0.5 mg/mL protein or 8.0 x 10^9^ OMV/mL in 5 fold serial dilutions. TLR7, TLR8, TLR9, NOD1 and NOD2 cell lines were challenged with 20 μL of OMVs in PBS starting at 1.0 mg/mL protein or 1.6 x 10^10^ OMV/mL in 2 fold serial dilutions. Positive controls for each cell line were used according to the manufacturer’s instructions. HEK-Blue Null cell controls were included in each experiment, challenged with OMVs to determine background alkaline phosphatase secretion. Following 20 hours of incubation 50 μL of supernatant was removed from each well and added to a clean 96-well plate with 150 μL of Quanti-Blue (Invivogen, USA). Alkaline phosphatase activity was determined at 620 nm on a spectrophotometer.

**REFERENCES**

Llorente A, S.T., Sylvänne T, Kauhanen D, Róg T, Orłowski A, Vattulainen I, Ekroos K, Sandvig K (2013). Molecular lipidomics of exosomes released by PC-3 prostate cancer cells. Biochim Biophys Acta *1831*, 1302-1309.

Olmstead ILD, H.D., Dias DA, Jayasinghe NS, Callahan DL, Kentish SE, Scales PJ, Martin GJO (2013). A quantitative analysis of microalgal lipids for optimization of biodiesel and omega-3 production. Biotechnology and Bioengineering *110*, 2096-2104.
