# Supplementary material for: Differential Responses of Pattern Recognition Receptors to Outer Membrane Vesicles of Three Periodontal Pathogens
Source: PLoS One. 2016 Apr 1;11(4):e0151967. doi: 10.1371/journal.pone.0151967 (PMC4818014; doi:10.1371/journal.pone.0151967)
Supplement: S1 Fig — (DOCX) [file pone.0151967.s002.docx]

**
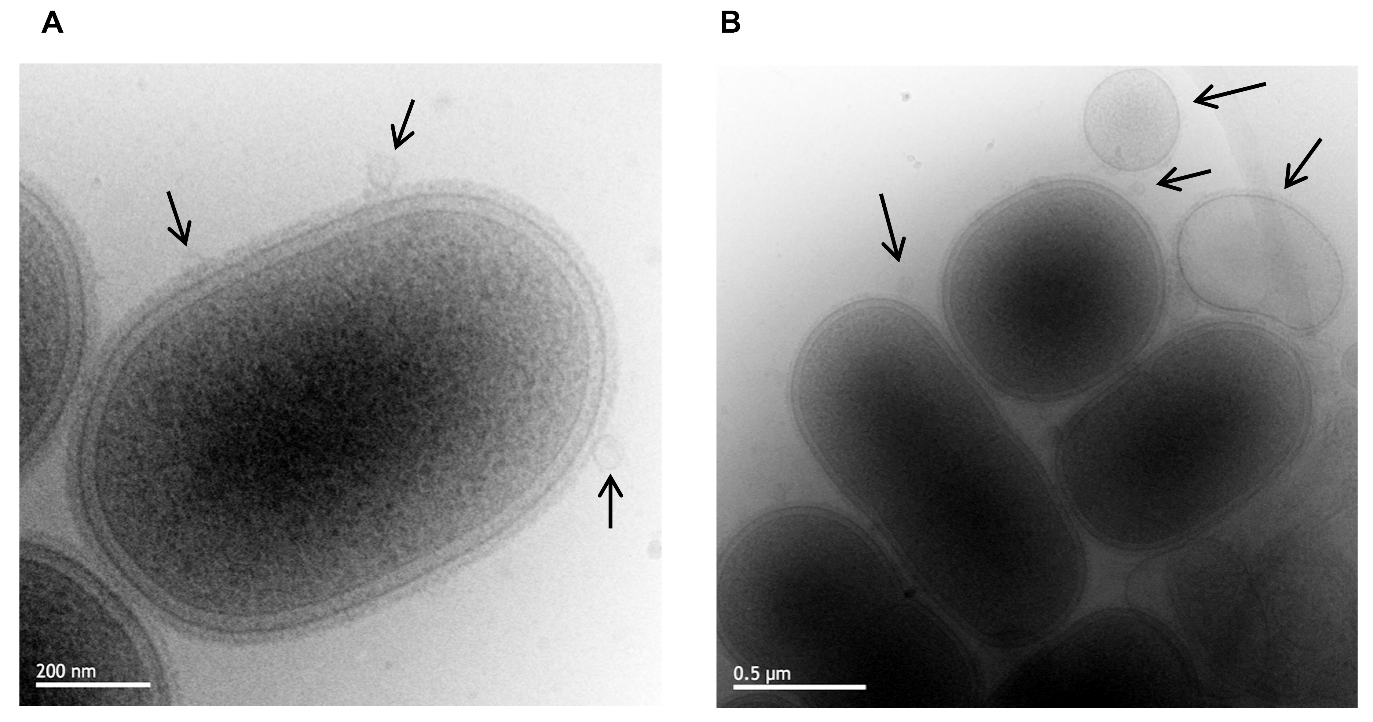
**

**S1 Fig. TEM of OMVs present in bacterial pellet.**

The *P. gingivalis* bacterial pellet was observed using transmission electron microscopy (TEM) (A B) to observe OMVs pelleting with bacteria (8000 g x 30 min) during initial removal from culture fluid.
