# Supplementary material for: Differential Responses of Pattern Recognition Receptors to Outer Membrane Vesicles of Three Periodontal Pathogens
Source: PLoS One. 2016 Apr 1;11(4):e0151967. doi: 10.1371/journal.pone.0151967 (PMC4818014; doi:10.1371/journal.pone.0151967)
Supplement: S2 Fig — (DOCX) [file pone.0151967.s003.docx]

*T. denticola*

Time (days)

OD

600

1

2

3

4

5

6

7

8

0.0

0.1

0.2

0.3

0.4

Filtered Serum

Unfiltered Serum

**A**

*T. forsythia*

Time (days)

OD

600

1

2

3

4

5

6

7

8

0.0

0.5

1.0

1.5

Filtered serum

Unfiltered Serum

**B**

**S2 Fig. Growth curve of *T. denticola* and *T. forsythia*.**

Growth curves of *T. denticola* ATCC 35405 (A) and *T. forsythia* ATCC 43037 (B) cultured in OBGM and TSBYK supplemented with unfiltered or 10kDa filtered heat inactivated Rabbit Serum and heat inactivated Fetal Calf Serum respectively. Cultures were grown for 8 days at 37 °C under anaerobic conditions. Optical Density was determined at 600nm.
