# Supplementary material for: Differential Responses of Pattern Recognition Receptors to Outer Membrane Vesicles of Three Periodontal Pathogens
Source: PLoS One. 2016 Apr 1;11(4):e0151967. doi: 10.1371/journal.pone.0151967 (PMC4818014; doi:10.1371/journal.pone.0151967)
Supplement: S3 Fig — (DOCX) [file pone.0151967.s004.docx]

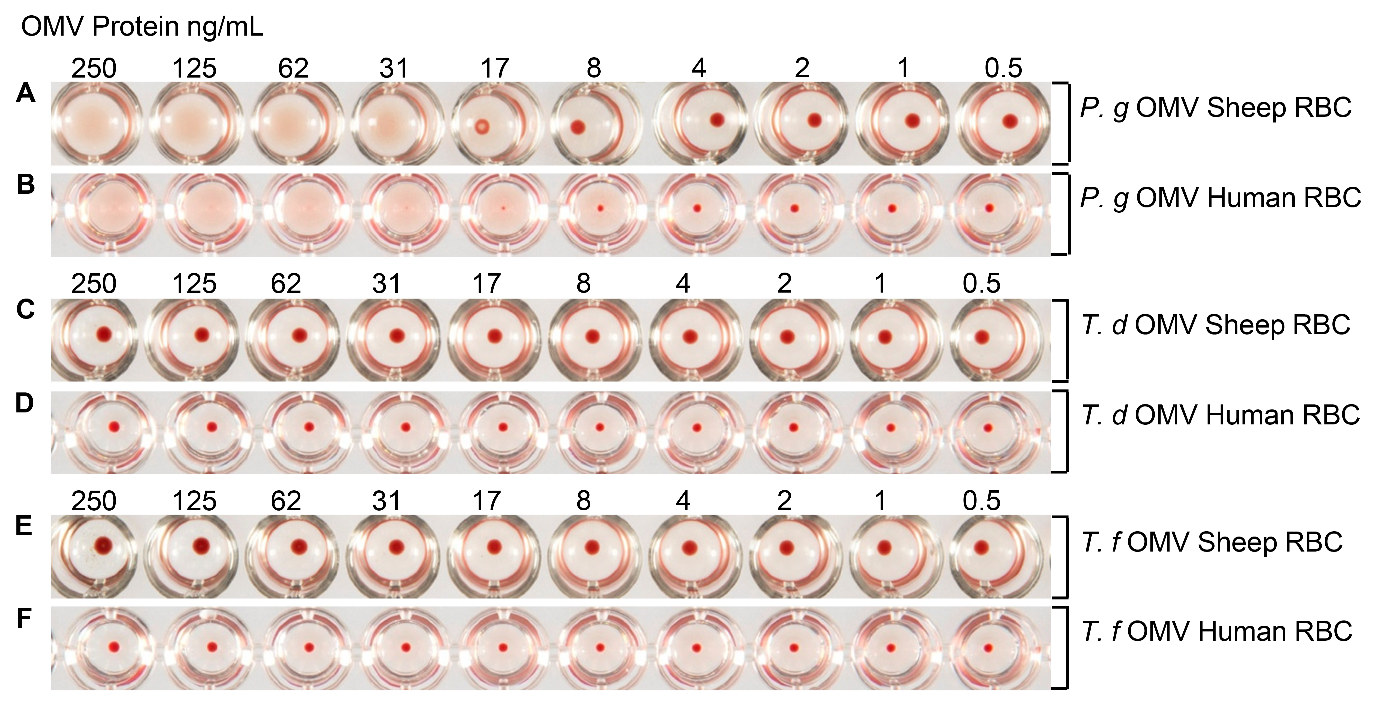


**S3 Fig. Haemagglutination activity of *P. gingivalis, T. denticola* and *T. forsythia* OMVs.**

Haemagglutination activity for *P. gingivalis* (A, B), *T. denticola* (C, D) and *T. forsythia* (E, F) purified OMVs was observed using both sheep and human red blood cells. OMV samples were diluted 2 fold across a round-bottomed 96 well plate. The titration endpoint was determined at the last well to see complete haemagglutination.
