# Supplementary material for: Differential Responses of Pattern Recognition Receptors to Outer Membrane Vesicles of Three Periodontal Pathogens
Source: PLoS One. 2016 Apr 1;11(4):e0151967. doi: 10.1371/journal.pone.0151967 (PMC4818014; doi:10.1371/journal.pone.0151967)
Supplement: S1 Table — (DOCX) [file pone.0151967.s005.docx]

**S1 Table. Protease Activity of *P. gingivalis*, *T. denticola* and *T. forsythia* OMVs**

|  | **BAPNA Activity ^a^ (U/mg/mL)** | **LYSNA Activity ^b^ (U/mg/mL)** |
| --- | --- | --- |
| ***P. gingivalis* OMV**  ***T. denticola* OMV**  ***T. forsythia* OMV** | 1.523 ± 0.065  0.015 ± 0.005  0.004 ± 0.001 | 0.765 ± 0.022  0.005 ± 0.001  0.001 ± 0.000 |

1. Arginine specific protease activity was determined for *P. gingivalis, T. denticola* and *T. forsythia* OMVs using protease substrate BAPNA. Results are presented as the rate of activity per mg protein per mL for each sample.
2. Lysine specific protease activity was determined for *P. gingiva*lis, *T. denticola* and *T. forsythia* OMVs using protease substrate LYSNA. Results are presented as the rate of activity per mg protein per mL for each sample.
